# *Tenebrio molitor* Larvae Meal Affects the Cecal Microbiota of Growing Pigs

**DOI:** 10.3390/ani10071151

**Published:** 2020-07-07

**Authors:** Sandra Meyer, Denise K. Gessner, Garima Maheshwari, Julia Röhrig, Theresa Friedhoff, Erika Most, Holger Zorn, Robert Ringseis, Klaus Eder

**Affiliations:** 1 Institute of Animal Nutrition and Nutrition Physiology, Justus Liebig University Giessen, Heinrich-Buff-Ring 26-32, 35392 Giessen, Germany; sandra.meyer@ernaehrung.uni-giessen.de (S.M.); denise.gessner@ernaehrung.uni-giessen.de (D.K.G.); Garima.Maheshwari@lcb.chemie.uni-giessen.de (G.M.); juliaroehrig@web.de (J.R.); theresa.friedhoff@ernaehrung.uni-giessen.de (T.F.); erika.most@ernaehrung.uni-giessen.de (E.M.); Klaus.Eder@ernaehrung.uni-giessen.de (K.E.); 2 Institute of Food Chemistry and Food Biotechnology, Justus Liebig University Giessen, Heinrich-Buff-Ring 17, 35392 Giessen, Germany; holger.zorn@lcb.chemie.uni-giessen.de; 3 Fraunhofer Institute for Molecular Biology and Applied Ecology, Winchester Str. 2, 35394 Giessen, Germany

**Keywords:** bacteroidetes, chitin, fermentation, firmicutes, intestine, insect meal, microbiota, pigs

## Abstract

**Simple Summary:**

Insect meal obtained from the mass-rearing of edible insects is increasingly considered as a potential alternative protein source in farm animal feeding, which can be produced with lower environmental impact than conventional protein sources, such as soybean meal—the currently main dietary protein source for monogastric farm animals. Apart from the necessity to overcome existing legal obstacles regarding the use of insect meal as feed for farm animals, a further prerequisite for the inclusion of insect meal in feeding rations for monogastric farm animals is that animals’ health is not impaired. Whether feeding insect meal to growing pigs alters gut microbiota composition, which is vital to both health and performance is currently unknown. The present study in growing pigs shows that dietary insect meal causes a characteristic shift in the cecal microbiota composition.

**Abstract:**

The hypothesis tested was that dietary inclusion of insect meal (IM) causes an alteration in the cecal microbiota composition and its fermentation activity of growing pigs. Five-week-old male crossbred pigs were randomly assigned to three groups of 10 pigs each, and fed isonitrogenous diets either without (CON) or with 5% IM (IM5) or 10% IM (IM10) from *Tenebrio molitor* larvae for four weeks. The relative abundance of the phylum Bacteroidetes was lower in group IM10 than in group CON (*p* < 0.05), whereas the relative abundance of Firmicutes and the Firmicutes:Bacteroidetes-ratio tended to be higher in groups IM10 and IM5 than in group CON (*p* < 0.1). The relative abundance of the Proteobacteria tended to be higher in group IM10 than in groups CON and IM5 (*p* < 0.1). The concentrations of the total short-chain fatty acids in the cecal digesta did not differ between the three groups, but the concentrations of the branched-chain fatty acids in the cecal digesta were higher in group IM5 and IM10 than in group CON (*p* < 0.05). The present study shows for the first time that the replacement of soybean meal by *Tenebrio molitor* larvae meal causes a shift of the cecal microbial community and its fermentation activity in growing pigs.

## 1. Introduction

Insect meal (IM) has been recognized as a promising alternative dietary protein source which can be produced with lower environmental impact than conventional protein sources, such as soybean extraction meal (SEM) [1]—the currently main dietary protein source for monogastric farm animals. Besides the high protein content of IM of approximately 70%, the high nutritive value of IM is explained by their good protein quality as evidenced by the fact that the concentrations of essential amino acids, with the exception of methionine, are comparable with those of animal proteins [2]. Apart from the necessity to overcome existing legal obstacles regarding the use of IM as feed for farm animals, a further prerequisite for the inclusion of IM in feeding rations for monogastric farm animals is that the animals´ health and performance is not impaired. While a significant number of studies in broilers indicate that IM from different edible insects is a suitable dietary protein source in this regard [3,4], few appropriate studies are available to reliably assess the suitability of IM as a protein source in feeding rations for pigs. We recently demonstrated by comprehensive analyses of the transcriptome, lipidome, and metabolome of key metabolic tissues that even complete replacement of SEM by IM from *Tenebrio molitor* larvae, which contained 70% crude protein, 9.8% crude lipids, and 5.2% crude ash (fresh matter) in the diet had no adverse effects on the metabolism of growing pigs [5]. This indicated the theoretic suitability of *Tenebrio molitor* larvae meal as a protein source in feeding rations for growing pigs.

However, our recent study in pigs revealed that the inclusion of a *Tenebrio molitor* larvae meal in the diets slightly, even though not significantly, decreased the body weight gain and feed efficiency of the pigs. This may be explained by the observation that the ileal digestibility of all amino acids was approximately 10%-units lower in pigs fed diets with *Tenebrio molitor* larvae meal than in pigs fed diets with SEM [5]. A decrease in the digestibility of nutrients, including protein in response to feeding IM, has also been observed in other monogastric animals [6,7] and is likely explained by the presence of the structural polysaccharide chitin in IM, an intrinsic constituent of the insects´ exoskeleton. The chitin content of the *Tenebrio molitor* larvae meal used in our recent study [5] was 9.2%. Like plant-derived non-starch polysaccharides (NSP), chitin is expected to encapsulate nutrients, thereby, acting as a physical barrier limiting nutrient digestibility. In addition, chitin in the intestinal digesta may increase, like NSP, digesta viscosity, which accelerates the transit of the intestinal digesta and increases the number of nutrients escaping digestion in the small intestine [8]. It has long been known that the microbiota of the large intestine utilizes nutrients escaping small intestinal digestion along with endogenous secretions (e.g., protein) as substrates for metabolism and growth. This explains that the microbiota composition and diversity in the large intestine is affected by changes in diet composition. In line with this, feeding diets containing *Tenebrio molitor* larvae meal was reported to alter microbiota composition and diversity in the cecum of broilers [9]. Whether feeding of *Tenebrio molitor* larvae meal to growing pigs also alters cecal microbiota composition and diversity is currently unknown. Alterations in the cecal microbiota of pigs might be of great relevance to pigs´ performance considering recent evidence that feed efficiency of pigs is strongly affected by the cecal microbiota through more complete digestion of substrates, an increased production of short-chain fatty acids (SCFA), or a decreased stimulation of the intestinal immune system [10,11].

Against this background, we hypothesized that inclusion of *Tenebrio molitor* larvae meal in the diet of growing pigs causes an alteration in the microbiota composition and the concentration of microbial fermentation products in the cecum. To test this hypothesis, cecal digesta and mucosa samples of pigs of our recent study [5], in which weaned piglets were fed isocaloric and isonitrogenous diets with either 0%, 5%, or 10% *Tenebrio molitor* larvae meal for four weeks, were used.

## 2. Materials and Methods

The study was approved by the local Animal Care and Use Committee (Regierungspräsidium Giessen; permission no: JLU 676_M). All experimental procedures described followed established guidelines for the care and handling of laboratory animals.

### 2.1. Animals and Diets

The experiment included weaned, male five-week-old crossbred pigs [Piétrain × (German Landrace × German Edelschwein)], which were randomly assigned to three groups of 10 pigs each [control (CON), 5% IM (IM5), 10% IM (IM10)], with similar initial BW of 8.69 ± 1.44 kg; mean ± SD; *N* = 30). In each treatment group, six pigs were kept in two pens of three animals and four pigs together in one pen under controlled conditions: 23 ± 2 °C ambient temperature, 50–60% relative humidity, light from 07:00 to 19:00. Three isoenergetic (based on gross energy content) and isonitrogenous experimental diets were fed, which met the nutrient requirements of growing pigs (German Society of Nutrition Physiology, [12]), but differed in their main protein source (Table 1). The diet in group CON contained SEM (44% crude protein/kg FM) as the main protein source. In the diets of groups IM5 and IM10, SEM was partially (50%) and completely (100%), respectively, replaced by *Tenebrio molitor* larvae meal (70% crude protein/kg FM) on an isonitrogenous basis. The analyzed content of crude nutrients, fatty acids, and amino acids of the *Tenebrio molitor* larvae meal are shown in Table 2. Cellulose was included in the diets of groups IM5 and IM10 at 2.8% and 5.7%, respectively, to ensure the isoenergetic replacement of SEM by *Tenebrio molitor* larvae meal. In order to cover the requirement of essential amino acids for growing pigs, the three diets were individually supplemented with different amounts of L-lysine, DL-methionine, L-threonine, L-tryptophan, and L-cysteine. Essential amino acids, which were contained in the diet components in sufficient amounts, were not supplemented. L-glutamic acid was added to the diets in different amounts to ensure that diets were isonitrogenous. All diets contained 0.5% titanium dioxide (TiO_2_) as an indicator allowing the calculation of the ileal digestibility of amino acids. Content of ether extract and main fatty acids of the three diets was adjusted by the addition of individual amounts of rapeseed oil and safflower oil to the diet of group CON and group IM5. The methods used to analyze the chemical composition of diets, fats, and *Tenebrio molitor* larvae meal have been described in our recent study [5], except the content of starch, which was determined according to official methods [13]. The content of metabolizable energy (ME) of the diets was calculated according to [14], using the following equations:

ME (MJ/kg DM) = 0.021503 × Crude protein (g/kg) + 0.032497 × Ether extract (g/kg) + 0.016309 × Starch (g/kg) + 0.014701 × Organic residue (g/kg) – 0.021071 × Crude fiber (g/kg)(1)

Organic residue = Dry matter – Crude ash – Crude protein – Ether extract − Starch(2)

The diets were offered *ad libitum* during the experimental period of four weeks. Water was constantly available *ad libitum* from a nipple drinker system.

### 2.2. Sample Collection

All pigs were killed by exsanguination under electronarcosis in the fed state. The gastrointestinal tract was removed, and the digesta from the cecum of all animals was collected. Fecal samples of all animals were obtained from the *Ampulla recti*. The small intestine was washed in ice-cold NaCl solution (0.9%) and opened lengthwise, and mucosa samples were collected from the jejunum (approximately 10 cm after the end of the duodenum) and ileum (approximately 5 cm prior to the ileal-cecal junction) by scraping, using a cell lifter (Santa Cruz Biotechnology, Dallas, TX, USA). Fecal samples, cecal digesta, and mucosa samples were snap-frozen in liquid nitrogen and stored at −80 °C pending analysis.

### 2.3. RNA Extraction and qPCR Analysis

Isolation of total RNA from small intestinal mucosa segments (approximately 20 mg) and assessment of RNA quantity and quality was carried out as described recently [15]. The average RNA concentration and the A260/A280 ratio of all the total RNA samples (*N* = 30, means ± SD) were 1.93 ± 0.31 µg/µL and 2.00 ± 0.02 (jejunal mucosa), and 1.68 ± 0.37 µg/µL and 2.00 ± 0.02 (ileal mucosa), respectively. Synthesis of cDNA and qPCR analysis was performed as described recently in detail [15]. Gene-specific primer pairs were designed to span an exon-exon junction using Primer3 [16] and Basic Local Alignment Search Tool (BLAST) [17] and synthesized by Eurofins MWG Operon (Ebersberg, Germany). The characteristics of primers are listed in Supplementary Table S1. The normalization was carried out using the procedure from Vandesompele et al. [18], according to which a normalization factor was calculated from the three most stable out of the multiple potential reference genes (*ATP5MC1*, *GAPDH*, *RPS9*, *SDHA*) tested. The three most stable reference genes were: *GAPDH*, *RPS9*, and *SDHA* (jejunum) and *ATP5MC1*, *GAPDH*, and *RPS9* (ileum).

### 2.4. Determination of Cecal Microbiota Composition and Diversity

Metagenomic DNA was isolated from approximately 180–200 mg of cecal digesta according to Lagkourvardos et al. [19] using genomic DNA columns (Macherey-Nagel, Düren, Germany) following the manufacturer’s instructions. The V3–V4 regions of the 16S rRNA genes were amplified using bacteria-specific primers following a two-step procedure according to the Illumina sequencing protocol, as described [19]. Amplicons were sequenced using a MiSeq system (Illumina, Inc., San Diego, CA, USA). The raw sequence data was processed using Integrated Microbial Next Generation Sequencing (IMNGS) [19]. Based on the UPARSE approach [19], raw sequences were demultiplexed, trimmed to the position of the first base (quality score < 3), and finally paired. Paired sequences with < 300 and > 600 nucleotides and expected error > 3 were further excluded. The remaining sequences were trimmed by 10 nucleotides on each end to avoid a GC bias and non-random base composition. Next, the presence of chimeras was checked using UCHIME [19]. Finally, sequences with a relative abundance > 0.5% in at least one sample were sorted, merged, and operational taxonomic units (OTU) were picked at a threshold of 97% similarity. Taxonomic classification to the OTU was assigned using the Ribosomal Database Project (RDP) classifier [20]. Further downstream analyses were done using Rhea (https://lagkouvardos.github.io/Rhea/) which uses the R programming environment (v. 3.6.3), including the R packages ade4 (v. 1.7-15) [21], GUniFrac (v. 1.1) [22], phangorn (v. 2.2.5) [23], Hmisc (v. 4.4-0) [24], plotrix (v. 3.7-8) [25], PerformanceAnalytics (v. 2.0.4) [26], reshape (v. 0.8.8) [27], ggplot2 (v. 3.3.2) [28], gridExtra (v. 2.3) [29], grid (v. 3.6.2) [30], ggrepel (v. 0.8.2) [31], gtable (v. 0.3.0) [32], and Matrix (v. 1.2-18) [33]. To compensate for the differential sequence depth between samples, OTU counts were normalized and relative abundances were calculated. The differential abundance analysis of taxa was performed on the aggregated data at the different taxonomic levels. For the evaluation of α-diversity among the samples, the Shannon and Simpson indices and their effective numbers were calculated using Rhea. To measure the similarity between the different microbial profiles, the β-diversity was determined by calculating the generalized UniFrac distances, as described previously [19]. Visualization of bacterial profiles among different groups was done by computation of non-metric multidimension distance matrix (NMDS) [34].

### 2.5. Determination of Concentrations of SCFA in Cecal Digesta of the Pigs

Cecal digesta SCFA concentrations were determined, as described previously [35]. In brief, 100 mg aliquots of cecal digesta were mixed with 1 mL 5% o-phosphoric acid containing an internal standard (0.3 mg/mL crotonic acid). Extraction was carried out by vortexing for 1 min, and the subsequent centrifugation at 21,000× g at 4 °C for 10 min. One microliter of the extract was injected into a gas chromatograph (Clarus 580 GC system, Perkin Elmer, Waltham, USA) equipped with a polar capillary column (10 m free fatty acid phase, 0.32 mm internal diameter, 0.25 μm film thickness; Macherey and Nagel, Düren, Germany) and a flame-ionization detector.

### 2.6. Determination of the Bile Acid Concentration in the Feces

To evaluate a potential effect of dietary treatment on bile acid output, the total bile acid concentration in the freeze-dried feces was measured using an enzymatic reagent kit (IDK Bile Acids Photometric test; Immundiagnostik, Bensheim, Germany).

### 2.7. Statistical Analysis

Statistical analyses were performed using the Minitab statistical software (Rel. 13.1, Minitab, State College, PA, USA). The experimental unit was the individual animal. Data were checked for distribution of normality by the Anderson–Darling test. Normally distributed data were analyzed by one-way ANOVA followed by Fisher’s multiple comparison test. If data were not normally distributed, the non-parametric Kruskal–Wallis test was used for inter-group comparison and the Bonferroni-corrected Mann–Whitney *U* test for between-group comparison. Differences were considered significant at *p* < 0.05. 

## 3. Results

### 3.1. Microbiota Diversity in the Cecum of the Pigs

**Figure 1:**
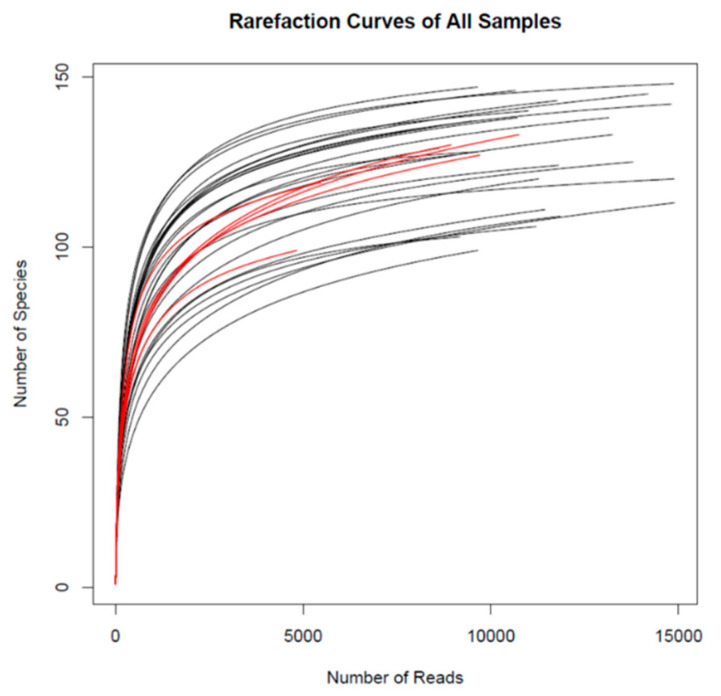
Rarefaction curves of all samples (*N* = 30). Red curves represent the samples with the lowest number of reads.

16S rRNA-based high-throughput sequencing revealed a total of 201 OTU in the pigs´ cecum which are listed in Supplementary Table S2. The number of sequences per sample after rarefaction was 10,794 ± 2540 (mean ± SD) in the average of all samples (*N* = 30). The rarefaction curves of all samples are shown in Figure 1, with red curves representing the samples with the lowest number of sequences.

**Figure 2:**
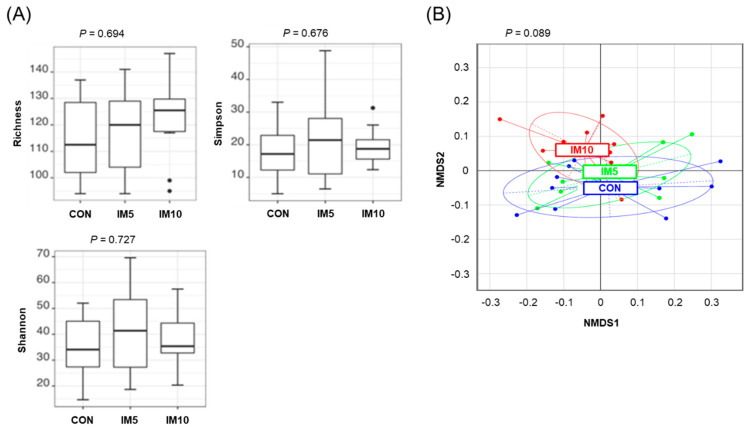
Effect of dietary treatment on cecal microbiota diversity in pigs fed isonitrogenous diets without (CON) or with 5% (IM5) or 10% *Tenebrio molitor larvae* meal (IM10) for four weeks. (**A**) Indicators of α-diversity (Richness, Simpson, Shannon) of the cecal bacterial community; (**B**) Visualization of the difference in β-diversity of cecal bacterial community between groups by meta non-metric multidimension distance matrix plot. The β-diversity of the cecal bacterial community was calculated based on generalized UniFrac distances.

The treatment effect on microbial diversity was evaluated by the use of different diversity metrics. The metrics used to describe α-diversity of the bacterial community in the cecum of the pigs did not differ between the groups (Figure 2A). The β-diversity of cecal bacterial community calculated based on generalized UniFrac distances, showed a tendency towards a difference between groups (*p* = 0.089). MetaNMDS plots were generated to visualize the difference in β-diversity of cecal bacterial community between groups (Figure 2B).

### 3.2. Microbiota Composition in the Cecum of the Pigs

**Figure 3:**
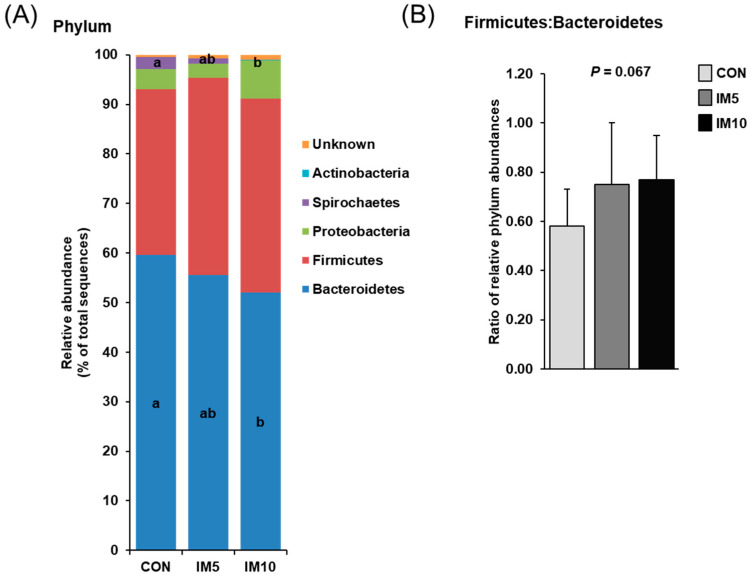
Effect of dietary treatment on cecal microbiota composition in pigs fed isonitrogenous diets without (CON) or with 5% (IM5) or 10% *Tenebrio molitor larvae* meal (IM10) for four weeks. (**A**) Distribution of cecal bacteria at the phylum level; (**B**) Ratio of the relative abundances of the two main phyla, Bacteroidetes, and Firmicutes. Values are means ± SD for *n* = 10 pigs per group. ^a,b^ Values with different superscripts differ significantly at *p* < 0.05.

Analysis of the cecal microbial community revealed that Bacteroidetes and Firmicutes represented the two most abundant phyla in the pigs´ cecum, together accounting for 90–95% of all bacteria in the three groups (Figure 3A). At the order level, the main bacteria in the pigs´ cecum of all groups were (in decreasing abundance) Bacteroidales, Clostridiales, Selenomonadales, Spirochaetales, Lactobacillales, Aeromonadales, and Bacillales (Table 3), with the first one belonging to the Bacteroidetes phylum and with Clostridiales, Selenomonadales, Lactobacillales, and Bacillales belonging to Firmicutes phylum. The relative abundance of the main phylum Bacteroidetes was lower in the cecum of group IM10 compared to group CON (*p* = 0.047, Figure 3A), although the relative abundance of its largest family, the Prevotellaceae, did not differ between groups. The relative abundance of Bacteroidetes in the cecum of group IM5 did not differ from that of group IM10 and group CON. The relative abundance of Firmicutes in the pigs´ cecum tended to be higher in groups IM10 and IM5 than in group CON (*p* = 0.078, Figure 3A). Within the Firmicutes, the predominant families were Lachnospiraceae, Ruminococcaceae, and Veillonellaceae in pigs´ cecum of all groups (Table 3). While the relative abundance of Lachnospiraceae was not different between groups, the relative abundances of Ruminococcaceae and Veillonellaceae in the cecum were lower and higher, respectively, in group IM10 than in groups IM5 and CON (*p* = 0.015 and *p* = 0.017, respectively). Owing to these changes in the two most abundant phyla, the Firmicutes:Bacteroidetes-ratio tended to be increased in groups IM10 and IM5 compared to group CON (*p* = 0.067, Figure 3B). The relative abundance of the third most abundant phylum Proteobacteria, which contributed 2.8–7.7% of all bacteria in the three groups tended to be higher in group IM10 than in groups CON and IM5 (*p* = 0.069, Figure 3A). The relative abundance of the Spirochaetes phylum, which made up only 0.1–2.4% of all bacteria in the three groups, and its main family Spirochaetaceae in the cecum was strongly reduced in group IM10 compared to group CON (*p* = 0.03 and *p* = 0.03, respectively, Figure 3A and Table 3), whereas group IM5 and group CON did not differ with regard to the abundance of this phylum. In contrast, the relative abundance of the least abundant phylum Actinobacteria and its main family Bifidobacteriaceae in cecal digesta was higher in group IM10 than in group CON (*p* = 0.002 and *p* = 0.002, respectively), whereas groups IM5 and CON were not different in this regard (Figure 3A).

At the genus level, the predominant genus in the cecal digesta of all groups was *Prevotella*, making up approximately 40–50% of all genera, followed by *Alloprevotella*, *Roseburia*, *Clostridium sensu stricto*, *Faecalibacterium*, *Lactobacillus*, *Treponema*, and *Phascolarctobacterium* (Table 3). Significant differences in the relative abundances of these genera between groups were found only for *Treponema* and some of the less abundant genera, *Bifidobacterium* and *Coprococcus*. While relative abundances of *Treponema* and *Coprococcus* in the pigs´ cecum were lower in group IM10 than in group CON (*p* = 0.03 and *p* = 0.011, respectively), the relative abundance of *Bifidobacterium* in the cecum was higher in group IM10 than in group CON (*p* = 0.002, Table 3). The relative abundance of *Streptococcus* in the cecum tended to be increased in group IM5 compared to group CON and group IM10 (*p* = 0.063, Table 3).

### 3.3. Concentrations of Microbial Fermentation Products in the Cecum of the Pigs

**Figure 4:**
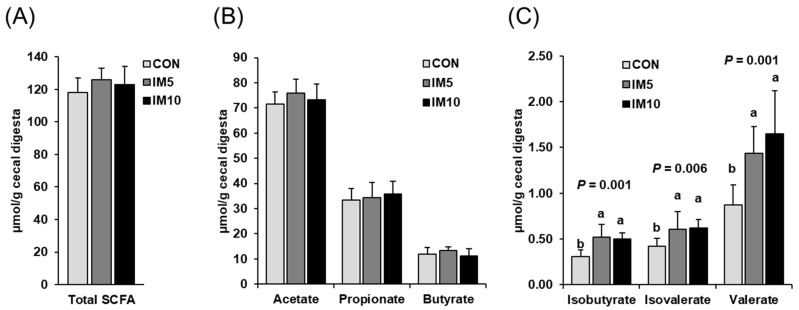
Concentrations of short-chain fatty acids (SCFA) in the cecal digesta of pigs fed isonitrogenous diets without (CON) or with 5% (IM5) or 10% *Tenebrio molitor larvae* meal (IM10) for four weeks. (**A**) total SCFA reflecting the sum of all SCFA detected; (**B**) individual main SCFA (acetate, propionate, butyrate); (**C**) individual minor SCFA (isobutyrate, isovalerate, valerate). Bars are means ± SD for *n* = 10 pigs per group. ^a,b^ Values with different superscripts differ significantly at *p* < 0.05.

Concentrations of total SCFA and individual main SCFA (acetic acid, propionic acid, butyric acid) in the cecal digesta did not differ between the three groups (Figure 4A,B). However, concentrations of the minor SCFA isobutyric acid, isovaleric acid, and valeric acid in cecal digesta were higher in group IM5 and IM10 than in group CON (*p* < 0.05, Figure 4C). Groups IM5 and IM10 did not differ with regard to the concentrations of the minor SCFA in cecal digesta.

### 3.4. Concentrations of Bile Acids in the Feces of the Pigs

**Figure 5:**
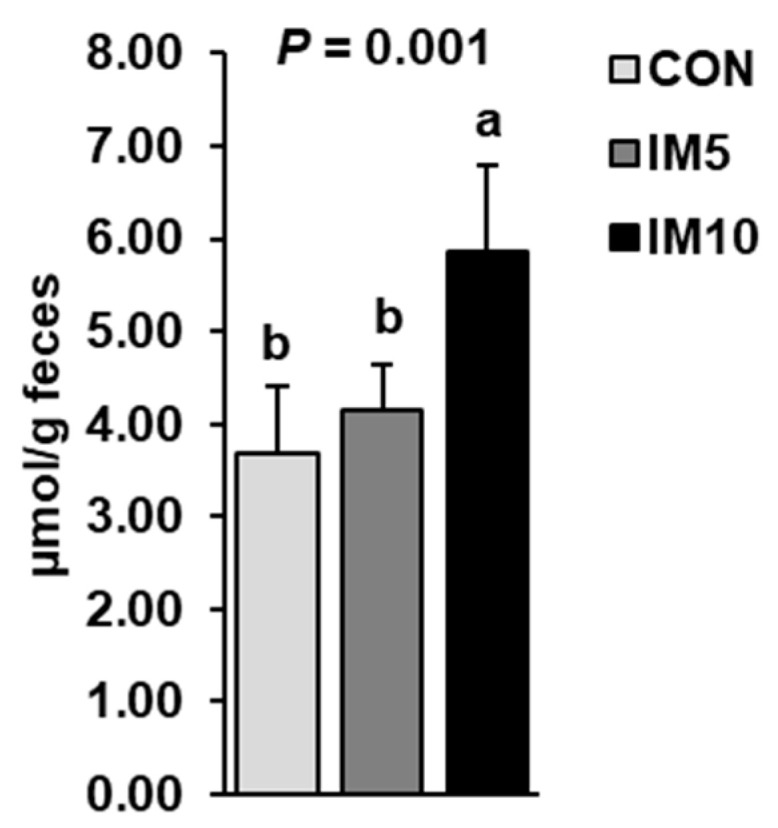
Concentrations of bile acids in the feces of pigs fed isonitrogenous diets without (CON) or with 5% (IM5) or 10% *Tenebrio molitor larvae* meal (IM10) for four weeks. Bars are means ± SD for *n* = 10 pigs per group. ^a,b^ Values with different superscripts differ significantly at *p* < 0.05.

The concentrations of total bile acids in the feces of the pigs were higher in group IM10 than in groups IM5 and CON (*p* = 0.001, Figure 5). Groups IM5 and CON did not differ in this regard.

### 3.5. Expression of Carbohydrate and Peptide Transporters in Small Intestinal Mucosa of the Pigs

**Figure 6:**
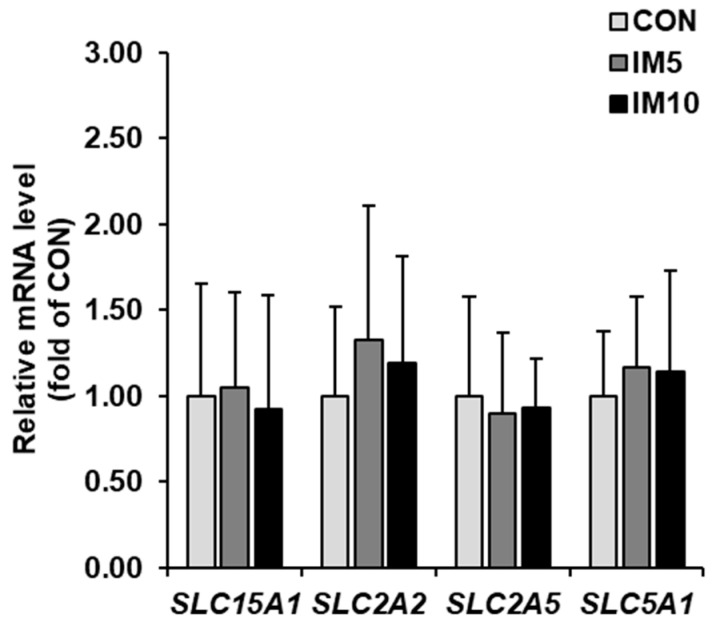
Relative mRNA levels of genes encoding nutrient transporters (solute carrier family 15 member 1 (*SLC15A1*), *SLC2A2*, *SLC2A5*, and *SLC5A1*) in the jejunal mucosa of pigs fed isonitrogenous diets without (CON) or with 5% (IM5) or 10% *Tenebrio molitor larvae* meal (IM10) for four weeks. Bars are means ± SD for *n* = 10 pigs per group. Relative mRNA levels are expressed as the fold of group CON (= 1.0). ^a,b^ Values with different superscripts differ significantly at *p* < 0.05.

The mRNA levels of genes encoding the nutrient transporters solute carrier family 15 member 1 (*SLC15A1*, formerly known as *PEPT1*), *SLC2A2* (formerly known as *GLUT2*), *SLC2A5* (formerly known as *GLUT5*), and *SLC5A1* (formerly known as *SGLT1*) in the jejunal mucosa did not differ between groups (Figure 6).

### 3.6. Expression of Genes Involved in Inflammation and Epithelial Barrier Function in Small Intestinal Mucosa of the Pigs

**Figure 7:**
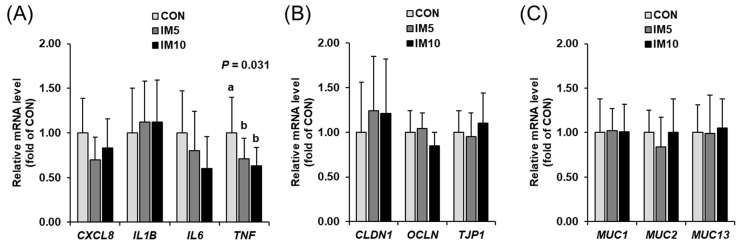
Relative mRNA levels of genes encoding inflammatory mediators (**A**: C-X-C motif chemokine ligand 8 (*CXCL8*), interleukin 1 beta (*IL1B*), *IL6*, and tumor necrosis factor (*TNF*)), tight junction proteins (**B**: claudin 1 (*CLDN1*), occludin (*OCLN*) and tight junction protein 1 (*TJP1*)), and mucins (**C**: mucin 1, cell surface-associated (*MUC1*), mucin 13, cell surface-associated (*MUC13*) and mucin 2, oligomeric mucus/gel-forming (*MUC2*)) in ileal mucosa of pigs fed isonitrogenous diets without (CON) or with 5% (IM5) or 10% *Tenebrio molitor larvae* meal (IM10) for four weeks. Bars are means ± SD for *n* = 10 pigs per group. Relative mRNA levels are expressed as fold of group CON (= 1.0). ^a,b^ Values with different superscripts differ significantly at *p* < 0.05.

While the mRNA levels of C-X-C motif chemokine ligand 8 (*CXCL8*) and interleukin 1 beta (*IL1B*) in ileal mucosa were not different between groups (Figure 7A), the mRNA level of tumor necrosis factor (*TNF*) was lower in groups IM5 and IM10 than in group CON (*p* = 0.031). The mRNA levels of genes encoding tight junction proteins [claudin 1 (*CLDN1*), occludin (*OCLN*), tight junction protein 1 (*TJP1*)], and mucins [mucin 1, cell surface-associated (*MUC1*), mucin 13, cell surface-associated (*MUC13*), mucin 2, oligomeric mucus/gel-forming (*MUC2*)] in ileal mucosa did not differ between the three groups (Figure 7B,C).

## 4. Discussion

In the present study, the hypothesis was tested that the inclusion of *Tenebrio molitor* larvae meal at the expense of SEM in the diet of growing pigs alters the cecal microbiota composition and its fermentation activity. In order to ensure isoenergetic replacement (based on gross energy content) of the crude protein of the diets, pure cellulose, which is one of the most prominent NSP in animal feed, was included in the IM5 and IM10 diets at levels of 2.8 and 5.7%, respectively. As a consequence of this, the crude fiber content of the diets increased from 6.3% (CON) to 8.0% (IM5) and 9.7% (IM10), respectively, and the ME content of diets slightly decreased from 14.6 MJ/kg DM (CON) to 14.1 MJ/kg DM (IM5), and 13.5 MJ/kg DM (IM10). Thus, the alterations in the cecal microbial community and its fermentation capacity induced by feeding the *Tenebrio molitor* larvae meal diets must be ascribed to some degree to the increased ingestion of cellulose and crude fiber. It is well-known that dietary cellulose and crude fiber exert nutrient-encapsulating effects and, thereby, limit nutrient digestion in the small intestine. Nonetheless, it is not unlikely that the effects on the cecal microbial community are partially caused by specific constituents of IM, such as chitin. A recent study in pigs demonstrated that dietary supplementation of prawn shell-derived chitosan, a chitin derivative with a low degree of acetylation, at a level of only 0.1% significantly alters the gut microbiota composition and decreases nutrient digestibility [36]. In the present study, the pigs´ diets in groups IM5 and IM10 contained approximately 0.5 and 1% chitin due to partial and complete replacement, respectively, of the crude protein of the diet. Although evidence exists that many monogastric animals including pigs secrete active chitinases in the stomach [37], the chitinolytic activity in the upper intestinal tract of pigs is likely limited allowing only partial digestion of chitin, and, thus, most of the chitin is expected to reach the large intestine, where it can be utilized as a fermentation substrate by specific cecal microbes, thus, causing an alteration of the cecal microbial community. In addition, despite the chitin content of the *Tenebrio molitor* larvae meal diets was relatively low, nutrient-encapsulating and also digesta viscosity-enhancing effects of chitin might have also contributed to a reduced protein digestibility in the small intestine of the pigs of group IM10 as observed recently [5]. In fact, decreased nutrient digestibility in response to feeding low levels (0.1%) of chitosan in pigs [36] and IM-containing diets in broilers and rats [6,7] has been reported. Supportive of a nutrient digestibility-depressing effect of chitin is also the observation that protein digestibility of chitin-free honey-bee protein concentrate was found to be higher than that of whole dried honey bees containing 11% chitin [38]. In addition, chitin but also crude fiber, are known for unspecific binding of sterols in the digesta or stimulation of bile acid excretion resulting in an increased fecal loss of bile acids [39,40]. Thus, particularly the higher crude fiber content, but also the chitin content, even though to a lesser extent, are likely causative for the finding that pigs of group IM10 had a higher concentration of bile acids in the feces than pigs of groups IM5 and CON.

To evaluate whether the inclusion of *Tenebrio molitor* larvae meal negatively affected the absorptive capacity of the small intestine of the pigs, the expression of main transporters for peptides and carbohydrates was determined in the mucosa of duodenum and jejunum, the two most important sites for peptide and carbohydrate absorption. Our observation that mRNA abundances of these transporters did not differ across the groups indicates that the inclusion of *Tenebrio molitor* larvae meal does not impair the absorptive capacity of the small intestine of pigs because the expression level of nutrient transporters in the small intestinal mucosa correlates with its absorptive capacity. The unaltered expression of nutrient transporters in the small intestinal mucosa of the pigs is in line with recent findings in other monogastric farm animals, like broilers, in which dietary inclusion of *Tenebrio molitor* meal also did not reduce the luminal absorptive area of the small intestine [3]. In addition, the inclusion of *Tenebrio molitor* larvae meal in the diets of groups IM5 and IM10 did not affect ileal mucosa expression of tight junction proteins and mucins, both of which are key elements of the intestinal barrier being critical for intestinal integrity and overall health of the organism [41]. In addition, the expression of the pro-inflammatory mediators in the ileal mucosa was either not altered (*CXCL8*, *IL1B*, *IL6*) or even reduced (*TNF*) by the *Tenebrio molitor* larvae meal, suggesting that the dietary inclusion of *Tenebrio molitor* larvae meal does not induce an inflammatory process in the intestinal mucosa which typically weakens the intestinal barrier function and also increases diversion of energy substrates and building blocks like amino acids away from growing tissues [42]. Overall, these findings indicate that the inclusion of *Tenebrio molitor* larvae meal in the diets had no negative impact on the integrity and functionality of the small intestine of the growing pigs.

With regard to the hypothesis of the present study, a key finding of this study was that the inclusion of *Tenebrio molitor* larvae meal in the diet, indeed, caused a significant alteration of the microbial community in the cecum of the pigs. The most striking alteration was a significant reduction in the abundance of Bacteroidetes, the most abundant phylum in the pigs´ cecum. This reduction was largely due to a decrease in the abundance of the main Bacteroidetes order Bacteroidales and its predominant family Prevotellaceae. By contrast, the present study revealed a tendency towards a higher abundance of Firmicutes, the second most abundant phylum in the cecum, in pigs fed *Tenebrio molitor* larvae meal diets. While the abundance of the main Firmicutes order Clostridiales did not differ across the groups, the abundance of the second most order of Firmicutes, the Selemonadales, and its main family Veillonellaceae was found to be increased in pigs fed IM. Owing to these changes in the two most abundant phyla, the Firmicutes:Bacteroidetes-ratio in cecal digesta tended to be increased in groups IM10 and IM5 compared to group CON. Similar alterations in the abundance of the two main bacterial phyla were also reported in the cecum of broilers fed *Tenebrio molitor* meal [9]. This suggests that the alteration of the cecal microbial community seen in the present study is characteristic for the use of *Tenebrio molitor* larvae meal in feeding rations for monogastric animals. Apart from alterations in the two main bacterial phyla, the inclusion of the high level of *Tenebrio molitor* larvae meal in the pigs´ diet caused a reduction in the abundance of the phylum Spirochaetes and its main genus *Treponema*, amongst which several pathogenic species are well documented [43], and an increase in the phylum Actinobacteria and its main family Bifidobacteriaceae in the cecum. The relevance of the alterations in these bacterial taxa, however, is unclear regarding their low relative abundance in the cecal bacterial community of the pigs. Nonetheless, due to the complex interplay between bacteria of different taxa within the microbial community, the *Tenebrio molitor* larvae meal-induced changes of low abundant taxa may also contribute to altered functionality of the gut microbiota, and thus, may affect host metabolism. Unlike microbial community composition, metrics of microbial diversity indicated either no effect (α-diversity) or only a tendency towards an effect (β-diversity) of inclusion of *Tenebrio molitor* larvae meal into the pigs´ feeding ration. In contrast, the diversity of the cecal microbiota in broilers was significantly increased by the dietary inclusion of *Tenebrio molitor* meal [9]. One reason for the lack of effect in the present study may be the relatively low number of animals used.

Owing to differences between bacteria of different taxa with regard to the metabolic pathways engaged in substrate utilization, a shift in the bacterial community may result in altered concentrations of microbiota-derived metabolites, such as SCFA, which are suitable indicators of the microbial fermentation activity. The finding that the concentrations of the total SCFA did not differ between control pigs and pigs fed *Tenebrio molitor* larvae meal suggests that the amounts of fermentable substrates reaching the large intestine were similar between groups, even though the type and the amounts of specific fermentable substrates (e.g., chitin, resistant starch, cellulose, protein) escaping digestion in the small intestine probably differed between the groups owing to differences in diet composition and its effect on digesta viscosity and digesta transit time. In addition, it suggests that the changes of the cecal bacterial community induced by the inclusion of the *Tenebrio molitor* larvae meal in the diet had no significant impact on the overall fermentation activity of the cecal microbiota of the pigs. Moreover, it was obvious that the proportions of the individual main SCFA (acetate, propionate, butyrate) did not differ between groups, despite the above-discussed alterations of the cecal bacterial community induced by the replacement of the crude protein in the diet. This is probably explained by the observation that the abundance of the dominant bacterial order Bacteroidales, whose members are known as acetate producers [44], tended to be decreased in pigs of group IM10, while the abundance of one of the main Firmicutes orders, the Selemonadales, which are also acetate producers [44], was increased in pigs fed *Tenebrio molitor* larvae meal diets. By contrast, the abundance of the dominating order within the Firmicutes, the Clostridiales, amongst which many bacterial families are typically butyrate producers [44], were not different between groups. Unlike the main SCFA, cecal concentrations of branched-chain fatty acids (isobutyrate, isovalerate) and valerate were increased in pigs of both *Tenebrio molitor* larvae meal-fed groups. While acetate, propionate, and butyrate are produced from fermentation of both, carbohydrates and proteins [45], the branched-chain fatty acids exclusively originate from fermentation of proteins, i.e., the branched amino acids valine, leucine, and isoleucine [46]. Thus, the increased cecal concentrations of isovalerate and isobutyrate may be reflective of a more pronounced protein fermentation in the large intestine of pigs fed *Tenebrio molitor* larvae meal diets, most likely, as a result of the abovementioned decrease of ileal digestibility of amino acids in the pigs. Similar findings have been reported in pigs fed diets containing chitin-oligosaccharides [47]. Despite an increased protein fermentation in the large intestine is regarded as detrimental for gut integrity and host´s health owing to increased formation of ammonia and other metabolites [48], certain fermentation products derived from aromatic amino acids, like indole, indole-3-aldehyde and indole-3-lactate, were even found to improve gut barrier function and reduce intestinal inflammation [49]. Even though not directly studied in the cecal mucosa, the unaltered expression of tight junction proteins, mucins and inflammatory mediators in ileal mucosa of pigs of groups IM5 and IM10 suggests that the *Tenebrio molitor* larvae meal-induced alterations of the cecal microbiota were not detrimental for cecal mucosa integrity because the ileal mucosa is also strongly exposed to the cecal microbes and its metabolites.

## 5. Conclusions

The present study shows that the inclusion of *Tenebrio molitor* larvae meal in the diet of growing pigs causes significant changes in the relative abundance of high-abundance (Bacteroidetes) and low-abundance bacterial taxa (Spirochaetes, Actinobacteria) and tends to increase the Firmicutes:Bacteroidetes-ratio in the cecum. Owing to the higher content of crude fiber in the *Tenebrio molitor* larvae meal-containing diets, the effect of *Tenebrio molitor* larvae meal on the cecal microbiota composition cannot be solely ascribed to specific constituents of *Tenebrio molitor* larvae meal such as chitin.

## Figures and Tables

**Table 1:** Composition, nutrient, and energy contents of the experimental diets.

Item	CON	IM5	IM10
*Components, g/kg*			
Wheat	380	380	380
Barley	250	250	250
Soybean meal (44% crude protein/kg FM)	147.8	73.9	-
*Tenebrio molitor* larvae meal (70% crude protein/kg FM)	-	50	100
Broad bean	110	110	110
Soybean oil	15.0	15.0	15.0
Rapseed oil	4.40	2.10	-
Safflower oil	3.00	1.60	-
Corn starch	3.90	3.00	1.50
Cellulose	-	28.0	56.5
Mineral and vitamin premix^1^	75.0	75.0	75.0
Monocalcium phosphate	6.00	6.00	6.00
Calcium carbonate	0.70	1.10	1.60
L-lysine	0.70	1.00	1.40
DL-methionine	-	0.10	0.10
L-threonine	0.20	0.10	-
L-tryptophan	0.10	0.10	0.20
L-glutamic acid	3.20	2.60	1.90
L-cysteine	-	0.40	0.80
Titanium dioxide	5.00	5.00	5.00
*Analyzed crude nutrient and energy content*			
Dry matter, % of FM	87.6	87.9	88.5
Crude protein, % of DM	22.2	22.7	22.8
Ether extract, % of DM	4.4	4.3	4.3
Crude ash, % of DM	5.8	5.4	5.0
Crude fiber, % of DM	6.3	8.0	9.7
Starch, % of DM	45.6	46.3	46.2
Gross energy, MJ/kg DM	19.5	19.3	19.6	
*Calculated energy content*			
Metabolizable energy, MJ/kg DM	14.6	14.1	13.5
*Analyzed amino acid content, g/kg DM*			
Alanine	7.8	9.1	9.5
Arginine	12.5	12.0	11.2
Aspartic acid	14.6	14.4	13.0
Cysteine	5.5	5.8	5.8
Glutamic acid	46.5	46.1	41.0
Glycine	8.2	8.6	8.7
Histidine	5.8	6.0	6.0
Isoleucine	8.6	8.9	8.7
Leucine	14.1	14.6	14.2
Lysine	13.7	15.8	15.3
Methionine	3.1	3.1	3.1
Phenylalanine	9.2	9.2	8.4
Proline	12.2	13.4	13.8
Serine	7.9	8.1	7.6
Taurine	3.7	3.7	3.6
Threonine	7.9	9.2	8.7
Tryptophan	2.9	2.9	3.0
Tyrosine	6.4	7.4	7.6
Valine	9.9	10.7	11.2
*Analyzed mineral content, g/kg DM*			
Calcium	9.9	9.6	9.5
Phosphorus	7.5	7.5	7.5

^1^ The premix provided the following minerals and vitamins (per kg diet): calcium, 6.75 g; phosphorus, 1.88 g; sodium 1.88; magnesium, 0.38 g; iron, 96 mg; copper, 150 mg; zinc, 75 mg; manganese, 75 mg; iodate, 2.25 mg; selenium, 0.45 mg; vitamin A, 21,000 IU; vitamin D_3_, 1875 IU; vitamin E, 150 mg; vitamin K, 3.75 mg; vitamin B1, 3.75 mg; vitamin B2, 10.5 mg; vitamin B6, 10.5 mg; vitamin B12, 75 µg; nicotinic acid, 52.5 mg; pantothenic acid, 26.3 mg; folic acid, 2.63 mg; biotin, 375 µg; choline, 375 mg; Abbreviations: DM, dry matter; FM, fresh matter.

**Table 2:** Analyzed content of crude nutrients, amino acids, and fatty acids of the *Tenebrio molitor* larvae meal.

Item	Insect Meal
*Crude nutrients*	
Crude protein, % of DM	74.0
Ether extract, % of DM	10.3
Crude fiber, % of DM	9.5
Crude ash, % of DM	5.5
Chitin, % of DM	9.7
Gross energy, MJ/kg DM	23.3
*Amino acids (g/kg DM)*	
Alanine	49.9
Arginine	34.4
Aspartic acid	61.2
Cysteine	5.7
Glutamic acid	86.8
Glycine	35.0
Histidine	18.1
Isoleucine	28.6
Leucine	51.2
Lysine	34.9
Methionine	8.1
Phenylalanine	24.2
Proline	57.5
Serine	31.2
Threonine	28.1
Tryptophan	7.9
Tyrosine	47.6
Valine	39.8
*Fatty acids^1^ (g/100 g total FAME)*	
12:0	0.3
14:0	2.4
16:0	15.5
16:1n-9	0.7
18:0	4.5
18:1n-9	35.0
18:2n-6	39.2
18:3n-3	1.5
20:0	0.1

^1^ Only fatty acids with concentrations of ≥ 0.1 g/100 g total fatty acids are shown; Abbreviations: DM, dry matter; FAME, fatty acid methyl esters; FM, fresh matter.

**Table 3:** Bacterial population composition (genus level) in the cecal digesta of pigs fed isonitrogenous diets without (CON) or with 5% (IM5) or 10% *Tenebrio molitor larvae* meal (IM10) for four weeks.

Classification Levels of Bacteria				CON	IM5	IM10	Pooled SD	*p*
Phylum	Order	Family	Genus					
Actinobacteria	Bifidobacteriales	Bifidobacteriaceae	Bifidobacterium	0.00 ^b^	0.02 ^ab^	0.09 ^a^	0.08	0.002
Bacteroidetes	Bacteroidales	Bacteroidaceae	Bacteroides	0.37	0.35	0.33	0.50	0.458
		Porphyromonadaceae	Barnesiella	0.30	0.30	0.38	0.20	0.407
			Parabacteroides	0.00	0.00	0.01	0.01	0.108
			unknown	0.73	0.34	0.73	0.72	0.450
		Prevotellaceae	Alloprevotella	6.42	7.53	8.08	2.26	0.208
			Prevotella	47.0	41.8	39.0	9.64	0.123
			unknown	3.15	3.89	2.47	2.26	0.662
		Rikenellaceae	Alistipes	0.00	0.00	0.02	0.02	0.080
		unknown		0.61	0.42	0.54	0.78	0.837
	unknown			1.00	0.91	0.45	0.95	0.772
Firmicutes	Bacillales	Bacillaceae 1	Bacillus	0.04	0.01	0.00	0.07	0.992
		Staphylococcaceae	Staphylococcus	0.02	0.01	0.00	0.03	0.372
	Lactobacillales	Lactobacillaceae	Lactobacillus	2.16	2.68	4.22	2.55	0.164
		Streptococcaceae	Streptococcus	0.05	0.09	0.03	0.11	0.063
	Clostridiales	Clostridiaceae 1	Clostridium sensu stricto	3.41	4.03	4.04	2.88	0.416
		Lachnospiraceae	Blautia	0.33	0.49	0.87	0.47	0.107
			Clostridium XlVa	0.33	0.40	0.45	0.32	0.661
			Coprococcus	0.10 ^a^	0.07 ^ab^	0.02 ^b^	0.06	0.011
			Fusicatenibacter	0.03	0.03	0.04	0.03	0.958
			Lachnospiracea incertae sedis	0.94	0.83	0.47	0.58	0.357
			Roseburia	4.17	4.98	3.68	1.73	0.276
			unknown	4.68	5.65	2.83	3.47	0.432
		Peptostreptococcaceae	Clostridium XI	0.57	0.90	0.92	0.53	0.327
		Ruminococcaceae	Clostridium IV	0.21	0.23	0.13	0.23	0.522
			Faecalibacterium	2.92	2.24	1.43	1.76	0.618
			Gemmiger	1.20	1.28	1.18	0.78	0.956
			Oscillibacter	0.68	0.62	0.68	0.41	0.953
			Ruminococcus	0.08	0.04	0.05	0.11	0.405
			unknown	2.68	3.73	2.42	2.42	0.547
		unknown		0.66	0.71	0.89	0.47	0.910
	Selenomonadales	Acidaminococcaceae	Phascolarctobacterium	1.64	1.87	2.11	0.86	0.257
		Veillonellaceae	Anaerovibrio	1.65	2.16	2.24	1.67	0.256
			Dialister	0.91	1.05	1.02	1.17	0.937
			Megasphaera	0.31	0.42	1.31	1.29	0.135
			Mitsuokella	0.73	1.05	2.38	1.71	0.239
			unknown	2.83	4.15	5.57	2.79	0.150
	unknown			0.18	0.21	0.14	0.24	0.887
Proteobacteria	unknown Betaproteobacteria			0.08	0.11	0.05	0.16	0.411
	unknown Deltaproteobacteria			0.63	0.32	0.47	0.52	0.371
	Aeromonadales	Succinivibrionaceae	Succinivibrio	1.16	0.77	1.44	1.01	0.657
	Enterobacteriales	Enterobacteriaceae	Escherichia/Shigella	0.04	0.02	0.49	0.64	0.317
	unknown Gammaproteobacteria			2.16	1.60	5.27	3.24	0.158
Spirochaetes	Spirochaetales	Spirochaetaceae	Treponema	2.38 ^a^	1.06 ^ab^	0.10 ^b^	2.65	0.030
Unknown				0.41	0.66	0.95		0.102

Values are means for *n* = 10 pigs per group; ^a,b^ Values within a row with different superscripts differ significantly at *p* < 0.05.

## Supplementary Materials

The following are available online at https://www.mdpi.com/2076-2615/10/7/1151/s1, Table S1: Characteristics of gene-specific primers used for qPCR analysis in the small intestinal mucosa, Table S2: Operational taxonomic units (OUT) identified in cecum digesta of all experimental groups.
