# Supplementary material for: Tenebrio molitor Larvae Meal Affects the Cecal Microbiota of Growing Pigs
Source: Animals (Basel). 2020 Jul 7;10(7):1151. doi: 10.3390/ani10071151 (PMC7401588; doi:10.3390/ani10071151)
Supplement: Supplementary file 1 [file animals-10-01151-s001.pdf]

Tenebrio Molitor Larvae Meal Affects the Cecal Microbiota of Growing Pigs

Sandra Meyer, Denise K. Gessner, Garima Maheshwari, Julia Röhrig, Theresa Friedhoff, Erika Most, Holger Zorn, Robert Ringseis and Klaus Eder

Table S1. Characteristics of gene-specific primers used for qPCR analysis in small intestinal mucosa.

Gene	Forward (5` to 3`)<br>Reverse (5` to 3`)	Annealing Temperature (°C)	PCR Product Size (bp)	NCBI GeneBank Accession No.	Slope	R^2	E
	Reference genes						
ATP5MC1	CAGTCACCTTGAGCCGGGCGA<br>TAGCGCCCCGGTGGTTTGC	64	94	NM_001025218	-3.55	0.999	1.91
GAPDH	AGGGGCTCTCCAGAACATCATCC<br>TCGCGTGCTCTTGCTGGGGTTGG	60	446	NM_001206359	-3.33	1.000	2.00
RPS9	GTCGCAAGACTTATGTGACC<br>AGCTTAAAGACCTGGGTCTG	62	325	XM_021094878	-3.28	0.999	2.02
SDHA	CTACGCCCCCGTCGCAAAGG<br>AGTTTGCCCCCAGGCGGTTG	64	380	XM_021076930	-3.32	1.000	2.00
	Target genes						
CLDN1	GATCGGCTCCATCGTCAGCA<br>CGACACGCAGGACATCCACA	60	115	NM_001244539	-3.54	0.994	1.92
CXCL8	ACTTCCAAACTGGCTGTTGC<br>GGAATGCGTATTTATGCACTGG	59	120	NM_213867	-3.59	0.994	1.90
IL1B	GTTCTCTGAGAAATGGGAGC<br>CTGGTCATCATCACAGAAGG	58	143	NM_214055	-3.51	0.997	1.93
IL6	AAGGTGATGCCACCTCAGAC<br>TCTGCCAGTACCTCCTTGCT	60	151	NM_001252429	-3.54	0.994	1.92
MUC1	CGGGCTTCTGGGACTCTTTT	58	312	XM_021089728	-3.63	0.999	1.89

	TTCTTTCGTCGGCACTGACA						
MUC13	TGTGTTTTGCTTTGGGTCCAG<br>CACAGCCAACTCCACTGTAGC	58	171	NM_001105293	-3.58	1.000	1.90
MUC2	CTTCCAACCATCCTCCCACC<br>GCCGTCTTGAAATCATCGCC	58	174	XM_021082584	-3.53	0.996	1.92
OCLN	GCCTACTCGTCCAACGGGAA<br>GCCCGTCGTGTAGTCTGTCT	60	246	NM_001163647	-3.60	0.999	1.90
SLC15A1	CAGACTTCGACCACAACGGA<br>TTATCCCGCCAGTACCCAGA	58	99	NM_214347	-3.52	0.998	1.93
SLC2A2	CGCAACCATTGGAGTTGGCGC<br>TGGCACAAACAAACATCCCACTCA	63	122	NM_001097417	-3.37	0.997	1.98
SLC2A5	CTGACACTGGTGCTTGCTTT<br>TTCGCTCATGTATTCCCCGA	57	156	XM_021095282	-3.45	0.989	1.95
SLC5A1	GTGGCGGACAGTAGTGAACA<br>AGAAGGCAGGATTTCAGGCA	57	89	NM_001164021	-3.31	1.000	1.92
TJP1	GTCGTCCTGATCCTGACCCG<br>TGGTGGGTTTGGTGGGTTGA	60	207	XM_021098827	-3.26	0.996	2.03
TNF	CCAAGGACTCAGATCATCGT<br>GCTGGTTGTCTTTCAGCTTC	58	146	NM_214022	-3.36	1.000	1.99

Table S2. Operational taxonomic units (OUT) identified in cecum digesta of all experimental groups.

OTU	Phylum	Class	Order	Family	Genus
OTU_1	Bacteroidetes	Bacteroidia	Bacteroidales	Prevotellaceae	Prevotella
OTU_2	Firmicutes	Clostridia	Clostridiales	Clostridiaceae 1	Clostridium sensu stricto
OTU_3	Firmicutes	Bacilli	Lactobacillales	Lactobacillaceae	Lactobacillus
OTU_4	Proteobacteria	Gammaproteobacteria	Orbales	Orbaceae	Frischella
OTU_5	Firmicutes	Negativicutes	Selenomonadales	Veillonellaceae	Anaerovibrio
OTU_6	Firmicutes	Negativicutes	Selenomonadales	Veillonellaceae	Anaerovibrio

OTU_7	Bacteroidetes	Bacteroidia	Bacteroidales	Prevotellaceae	Prevotella
OTU_8	Firmicutes	Clostridia	Clostridiales	Lachnospiraceae	Roseburia
OTU_9	Bacteroidetes	Bacteroidia	Bacteroidales	Prevotellaceae	Prevotella
OTU_10	Bacteroidetes	Bacteroidia	Bacteroidales	Prevotellaceae	Alloprevotella
OTU_11	Firmicutes	Negativicutes	Selenomonadales	Acidaminococcaceae	Phascolarctobacterium
OTU_12	Proteobacteria	Gammaproteobacteria	Aeromonadales	Succinivibrionaceae	Succinivibrio
OTU_13	Firmicutes	Clostridia	Clostridiales	Ruminococcaceae	Faecalibacterium
OTU_14	Firmicutes	Negativicutes	Selenomonadales	Veillonellaceae	Anaerovibrio
OTU_15	Firmicutes	Negativicutes	Selenomonadales	Veillonellaceae	Dialister
OTU_16	Spirochaetes	Spirochaetia	Spirochaetales	Spirochaetaceae	Treponema
OTU_17	Bacteroidetes	Bacteroidia	Bacteroidales	Prevotellaceae	Prevotella
OTU_18	Firmicutes	Clostridia	Clostridiales	Ruminococcaceae	Gemmiger
OTU_19	Firmicutes	Clostridia	Clostridiales	Peptostreptococcaceae	Terrisporobacter
OTU_20	Bacteroidetes	Bacteroidia	Bacteroidales	Prevotellaceae	Prevotella
OTU_21	Firmicutes	Clostridia	Clostridiales	Lachnospiraceae	Roseburia
OTU_22	Firmicutes	Negativicutes	Selenomonadales	Veillonellaceae	Megasphaera
OTU_23	Bacteroidetes	Bacteroidia	Bacteroidales	Prevotellaceae	Alloprevotella
OTU_24	Bacteroidetes	Bacteroidia	Bacteroidales	Prevotellaceae	Prevotella
OTU_25	Bacteroidetes	Bacteroidia	Bacteroidales	Bacteroidaceae	Anaerorhabdus
OTU_26	Firmicutes	Negativicutes	Selenomonadales	Veillonellaceae	Mitsuokella
OTU_27	Firmicutes	Clostridia	Clostridiales	Ruminococcaceae	Oscillibacter
OTU_28	Firmicutes	Clostridia	Clostridiales	Lachnospiraceae	Lachnospiracea_incertae_sedis
OTU_29	Firmicutes	Clostridia	Clostridiales	Lachnospiraceae	Lachnospiracea_incertae_sedis
OTU_30	Firmicutes	Clostridia	Clostridiales	Ruminococcaceae	Gemmiger
OTU_31	Bacteroidetes	Bacteroidia	Bacteroidales	Prevotellaceae	Alloprevotella
OTU_32	Bacteroidetes	Bacteroidia	Bacteroidales	Prevotellaceae	Paraprevotella
OTU_33	Bacteroidetes	Bacteroidia	Bacteroidales	Prevotellaceae	Paraprevotella
OTU_34	Firmicutes	Clostridia	Clostridiales	Lachnospiraceae	Clostridium XlVa
OTU_35	Bacteroidetes	Bacteroidia	Bacteroidales	Prevotellaceae	Prevotella
OTU_37	Firmicutes	Clostridia	Clostridiales	Ruminococcaceae	Gemmiger
OTU_38	Bacteroidetes	Bacteroidia	Bacteroidales	Prevotellaceae	Prevotella
OTU_39	Bacteroidetes	Bacteroidia	Bacteroidales	Prevotellaceae	Alloprevotella
OTU_40	Bacteroidetes	Bacteroidia	Bacteroidales	Porphyromonadaceae	Falsiporphyromonas

OTU_41	Bacteroidetes	Bacteroidia	Bacteroidales	Prevotellaceae	Prevotella
OTU_42	Spirochaetes	Spirochaetia	Spirochaetales	Spirochaetaceae	Treponema
OTU_43	Bacteroidetes	Bacteroidia	Bacteroidales	Prevotellaceae	Prevotella
OTU_44	Bacteroidetes	Bacteroidia	Bacteroidales	Prevotellaceae	Prevotella
OTU_45	Firmicutes	Clostridia	Clostridiales	Ruminococcaceae	Faecalibacterium
OTU_46	Firmicutes	Clostridia	Clostridiales	Lachnospiraceae	Blautia
OTU_47	Bacteroidetes	Bacteroidia	Bacteroidales	Prevotellaceae	Prevotella
OTU_48	Bacteroidetes	Bacteroidia	Bacteroidales	Prevotellaceae	Prevotella
OTU_49	Bacteroidetes	Bacteroidia	Bacteroidales	Prevotellaceae	Prevotella
OTU_50	Bacteroidetes	Bacteroidia	Bacteroidales	Porphyromonadaceae	Macellibacteroides
OTU_51	Bacteroidetes	Bacteroidia	Bacteroidales	Prevotellaceae	Prevotella
OTU_52	Firmicutes	Clostridia	Clostridiales	Lachnospiraceae	Coprococcus
OTU_54	Proteobacteria	Gammaproteobacteria	Aeromonadales	Succinivibrionaceae	Succinivibrio
OTU_55	Firmicutes	Clostridia	Clostridiales	Ruminococcaceae	Sporobacter
OTU_56	Firmicutes	Clostridia	Clostridiales	Peptostreptococcaceae	Clostridium XI
OTU_57	Bacteroidetes	Bacteroidia	Bacteroidales	Prevotellaceae	Prevotella
OTU_58	Proteobacteria	Deltaproteobacteria	Bdellovibrionales	Bdellovibrionaceae	Vampirovibrio
OTU_59	Spirochaetes	Spirochaetia	Spirochaetales	Spirochaetaceae	Treponema
OTU_60	Bacteroidetes	Bacteroidia	Bacteroidales	Marinilabiliaceae	Alkalitalea
OTU_61	Proteobacteria	Gammaproteobacteria	Enterobacteriales	Enterobacteriaceae	Escherichia/Shigella
OTU_62	Firmicutes	Clostridia	Clostridiales	Ruminococcaceae	Clostridium IV
OTU_63	Bacteroidetes	Bacteroidia	Bacteroidales	Prevotellaceae	Paraprevotella
OTU_65	Firmicutes	Bacilli	Lactobacillales	Lactobacillaceae	Lactobacillus
OTU_66	Bacteroidetes	Bacteroidia	Bacteroidales	Prevotellaceae	Prevotella
OTU_67	Firmicutes	Clostridia	Clostridiales	Lachnospiraceae	Lachnospiracea_incertae_sedis
OTU_68	Bacteroidetes	Bacteroidia	Bacteroidales	Prevotellaceae	Alloprevotella
OTU_69	Firmicutes	Clostridia	Clostridiales	Lachnospiraceae	Blautia
OTU_70	Firmicutes	Negativicutes	Selenomonadales	Veillonellaceae	Anaerovibrio
OTU_71	Bacteroidetes	Bacteroidia	Bacteroidales	Prevotellaceae	Prevotella
OTU_72	Firmicutes	Clostridia	Clostridiales	Ruminococcaceae	Flavonifractor
OTU_73	Firmicutes	Clostridia	Clostridiales	Eubacteriaceae	Eubacterium
OTU_74	Bacteroidetes	Bacteroidia	Bacteroidales	Prevotellaceae	Alloprevotella
OTU_75	Firmicutes	Clostridia	Clostridiales	Lachnospiraceae	Lachnospiracea_incertae_sedis

OTU_76	Firmicutes	Clostridia	Clostridiales	Lachnospiraceae	Lachnospiracea_incertae_sedis
OTU_77	Bacteroidetes	Bacteroidia	Bacteroidales	Prevotellaceae	Prevotella
OTU_79	Firmicutes	Clostridia	Clostridiales	Ruminococcaceae	Clostridium IV
OTU_80	Bacteroidetes	Bacteroidia	Bacteroidales	Porphyromonadaceae	Falsiporphyromonas
OTU_81	Bacteroidetes	Bacteroidia	Bacteroidales	Bacteroidaceae	Bacteroides
OTU_82	Firmicutes	Clostridia	Clostridiales	Lachnospiraceae	Butyrivibrio
OTU_83	Firmicutes	Clostridia	Clostridiales	Clostridiaceae 1	Clostridium sensu stricto
OTU_84	Firmicutes	Clostridia	Clostridiales	Ruminococcaceae	Anaerobacterium
OTU_85	Firmicutes	Clostridia	Clostridiales	Lachnospiraceae	Roseburia
OTU_86	Firmicutes	Bacilli	Lactobacillales	Lactobacillaceae	Lactobacillus
OTU_88	Firmicutes	Clostridia	Clostridiales	Lachnospiraceae	Clostridium XlVa
OTU_91	Firmicutes	Clostridia	Clostridiales	Ruminococcaceae	Flavonifractor
OTU_92	Firmicutes	Clostridia	Clostridiales	Clostridiaceae 1	Clostridium sensu stricto
OTU_93	Firmicutes	Clostridia	Clostridiales	Christensenellaceae	Christensenella
OTU_94	Bacteroidetes	Bacteroidia	Bacteroidales	Prevotellaceae	Paraprevotella
OTU_95	Bacteroidetes	Bacteroidia	Bacteroidales	Porphyromonadaceae	Barnesiella
OTU_97	Bacteroidetes	Bacteroidia	Bacteroidales	Prevotellaceae	Prevotella
OTU_99	Bacteroidetes	Bacteroidia	Bacteroidales	Prevotellaceae	Prevotella
OTU_100	Firmicutes	Clostridia	Clostridiales	Lachnospiraceae	Lactonifactor
OTU_101	Firmicutes	Bacilli	Lactobacillales	Streptococcaceae	Streptococcus
OTU_105	Bacteroidetes	Bacteroidia	Bacteroidales	Prevotellaceae	Prevotella
OTU_108	Bacteroidetes	Bacteroidia	Bacteroidales	Porphyromonadaceae	Barnesiella
OTU_110	Firmicutes	Clostridia	Clostridiales	Ruminococcaceae	Sporobacter
OTU_112	Bacteroidetes	Bacteroidia	Bacteroidales	Prevotellaceae	Prevotella
OTU_113	Proteobacteria	Deltaproteobacteria	Bdellovibrionales	Bdellovibrionaceae	Vampirovibrio
OTU_115	Bacteroidetes	Bacteroidia	Bacteroidales	Prevotellaceae	Prevotella
OTU_116	Firmicutes	Clostridia	Clostridiales	Clostridiales_Incertae Sedis XII	Guggenheimella
OTU_118	Bacteroidetes	Bacteroidia	Bacteroidales	Porphyromonadaceae	Barnesiella
OTU_119	Firmicutes	Clostridia	Clostridiales	Lachnospiraceae	Clostridium XlVa
OTU_122	Firmicutes	Clostridia	Clostridiales	Lachnospiraceae	Lachnospiracea_incertae_sedis
OTU_126	Bacteroidetes	Bacteroidia	Bacteroidales	Prevotellaceae	Prevotella
OTU_127	Firmicutes	Negativicutes	Selenomonadales	Veillonellaceae	Selenomonas

OTU_130	Firmicutes	Clostridia	Clostridiales	Lachnospiraceae	Clostridium XlVa
OTU_131	Proteobacteria	Betaproteobacteria	Rhodocyclales	Rhodocyclaceae	Azovibrio
OTU_132	Firmicutes	Clostridia	Clostridiales	Lachnospiraceae	Lachnospiracea_incertae_sedis
OTU_134	Firmicutes	Clostridia	Clostridiales	Ruminococcaceae	Ruminococcus
OTU_142	Bacteroidetes	Bacteroidia	Bacteroidales	Prevotellaceae	Prevotella
OTU_145	Bacteroidetes	Bacteroidia	Bacteroidales	Prevotellaceae	Prevotella
OTU_147	Firmicutes	Clostridia	Clostridiales	Lachnospiraceae	Clostridium XlVa
OTU_151	Bacteroidetes	Bacteroidia	Bacteroidales	Bacteroidaceae	Bacteroides
OTU_153	Firmicutes	Clostridia	Clostridiales	Ruminococcaceae	Papillibacter
OTU_154	Firmicutes	Clostridia	Clostridiales	Ruminococcaceae	Clostridium IV
OTU_157	Firmicutes	Negativicutes	Selenomonadales	Veillonellaceae	Selenomonas
OTU_158	Firmicutes	Clostridia	Clostridiales	Ruminococcaceae	Ruminococcus
OTU_159	Firmicutes	Clostridia	Clostridiales	Ruminococcaceae	Oscillibacter
OTU_161	Actinobacteria	Actinobacteridae	Bifidobacteriales	Bifidobacteriaceae	Bifidobacteriaceae
OTU_163	Bacteroidetes	Bacteroidia	Bacteroidales	Prevotellaceae	Prevotella
OTU_166	Firmicutes	Clostridia	Clostridiales	Ruminococcaceae	Oscillibacter
OTU_172	Firmicutes	Clostridia	Clostridiales	Lachnospiraceae	Clostridium XlVa
OTU_175	Firmicutes	Clostridia	Clostridiales	Lachnospiraceae	Clostridium XlVa
OTU_184	Firmicutes	Negativicutes	Selenomonadales	Veillonellaceae	Mitsuokella
OTU_188	Bacteroidetes	Bacteroidia	Bacteroidales	Porphyromonadaceae	Macellibacteroides
OTU_193	Firmicutes	Clostridia	Clostridiales	Lachnospiraceae	Roseburia
OTU_198	Firmicutes	Clostridia	Clostridiales	Lachnospiraceae	Fusicatenibacter
OTU_218	Bacteroidetes	Bacteroidia	Bacteroidales	Prevotellaceae	Paraprevotella
OTU_226	Firmicutes	Clostridia	Clostridiales	Lachnospiraceae	Butyrivibrio
OTU_235	Firmicutes	Clostridia	Clostridiales	Ruminococcaceae	Intestinimonas
OTU_240	Bacteroidetes	Bacteroidia	Bacteroidales	Prevotellaceae	Prevotella
OTU_247	Bacteroidetes	Bacteroidia	Bacteroidales	Bacteroidaceae	Bacteroides
OTU_272	Firmicutes	Clostridia	Clostridiales	Ruminococcaceae	Oscillibacter
OTU_276	Firmicutes	Clostridia	Clostridiales	Ruminococcaceae	Clostridium IV
OTU_282	Bacteroidetes	Bacteroidia	Bacteroidales	Porphyromonadaceae	Acetobacteroides
OTU_321	Firmicutes	Clostridia	Clostridiales	Clostridiales_Incertae Sedis XI	Dethiosulfatibacter
OTU_330	Firmicutes	Clostridia	Clostridiales	Lachnospiraceae	Coprococcus

OTU_333	Bacteroidetes	Bacteroidia	Bacteroidales	Porphyromonadaceae	Barnesiella
OTU_346	Bacteroidetes	Bacteroidia	Bacteroidales	Bacteroidaceae	Bacteroides
OTU_347	Bacteroidetes	Bacteroidia	Bacteroidales	Prevotellaceae	Alloprevotella
OTU_352	Firmicutes	Clostridia	Clostridiales	Lachnospiraceae	Clostridium XlVa
OTU_353	Firmicutes	Bacilli	Bacillales	Bacillaceae 1	Bacillus
OTU_363	Bacteroidetes	Bacteroidia	Bacteroidales	Prevotellaceae	Alloprevotella
OTU_365	Bacteroidetes	Bacteroidia	Bacteroidales	Porphyromonadaceae	Parabacteroides
OTU_367	Bacteroidetes	Bacteroidia	Bacteroidales	Prevotellaceae	Prevotella
OTU_383	Firmicutes	Clostridia	Clostridiales	Ruminococcaceae	Flavonifractor
OTU_391	Bacteroidetes	Bacteroidia	Bacteroidales	Bacteroidaceae	Bacteroides
OTU_394	Bacteroidetes	Bacteroidia	Bacteroidales	Rikenellaceae	Alistipes
OTU_396	Firmicutes	Bacilli	Bacillales	Staphylococcaceae	Staphylococcus
OTU_417	Bacteroidetes	Bacteroidia	Bacteroidales	Bacteroidaceae	Bacteroides
OTU_418	Bacteroidetes	Bacteroidia	Bacteroidales	Marinilabiliaceae	Anaerophaga
OTU_441	Firmicutes	Clostridia	Clostridiales	Lachnospiraceae	Clostridium XlVa
OTU_446	Bacteroidetes	Bacteroidia	Bacteroidales	Rikenellaceae	Alistipes
OTU_457	Bacteroidetes	Bacteroidia	Bacteroidales	Rikenellaceae	Alistipes
OTU_463	Proteobacteria	Betaproteobacteria	Burkholderiales	Sutterellaceae	Sutterella
OTU_465	Firmicutes	Clostridia	Clostridiales	Lachnospiraceae	Clostridium XlVa
OTU_467	Proteobacteria	Gammaproteobacteria	Pseudomonadales	Pseudomonadaceae	Pseudomonas
OTU_478	Firmicutes	Clostridia	Clostridiales	Clostridiaceae 1	Clostridium sensu stricto
OTU_482	Firmicutes	Clostridia	Clostridiales	Lachnospiraceae	Acetatifactor
OTU_501	Bacteroidetes	Bacteroidia	Bacteroidales	Bacteroidaceae	Bacteroides
OTU_523	Firmicutes	Clostridia	Clostridiales	Christensenellaceae	Christensenella
OTU_544	Bacteroidetes	Bacteroidia	Bacteroidales	Porphyromonadaceae	Parabacteroides
OTU_561	Firmicutes	Clostridia	Clostridiales	Lachnospiraceae	Clostridium XlVa
OTU_562	Firmicutes	Clostridia	Clostridiales	Ruminococcaceae	Flavonifractor
OTU_573	Bacteroidetes	Bacteroidia	Bacteroidales	Prevotellaceae	Alloprevotella
OTU_599	Actinobacteria	Actinobacteria	Actinobacteridae	Actinomycetales	Micrococcineae
OTU_607	Proteobacteria	Deltaproteobacteria	Bdellovibrionales	Bdellovibrionaceae	Vampirovibrio
OTU_663	Firmicutes	Clostridia	Clostridiales	Ruminococcaceae	Sporobacter
OTU_669	Firmicutes	Clostridia	Clostridiales	Lachnospiraceae	Coprococcus
OTU_670	Firmicutes	Bacilli	Lactobacillales	Enterococcaceae	Enterococcus

OTU_673	Actinobacteria	Actinobacteria	Coriobacteriales	Coriobacteriaceae	Enterorhabdus
OTU_683	Bacteroidetes	Bacteroidia	Bacteroidales	Prevotellaceae	Prevotella
OTU_703	Proteobacteria	Deltaproteobacteria	Bdellovibrionales	Bdellovibrionaceae	Vampirovibrio
OTU_717	Firmicutes	Negativicutes	Selenomonadales	Veillonellaceae	Mitsuokella
OTU_728	Firmicutes	Clostridia	Clostridiales	Ruminococcaceae	Faecalibacterium
OTU_731	Bacteroidetes	Bacteroidia	Bacteroidales	Rikenellaceae	Alistipes
OTU_738	Firmicutes	Clostridia	Clostridiales	Lachnospiraceae	Ruminococcus2
OTU_740	Firmicutes	Clostridia	Clostridiales	Lachnospiraceae	Hungatella
OTU_746	Bacteroidetes	Bacteroidia	Bacteroidales	Rikenellaceae	Alistipes
OTU_751	Firmicutes	Clostridia	Clostridiales	Ruminococcaceae	Clostridium IV
OTU_761	Firmicutes	Clostridia	Clostridiales	Lachnospiraceae	Ruminococcus2
OTU_805	Firmicutes	Clostridia	Clostridiales	Lachnospiraceae	Ruminococcus2
OTU_806	Bacteroidetes	Bacteroidia	Bacteroidales	Rikenellaceae	Alistipes
OTU_886	Proteobacteria	Gammaproteobacteria	Aeromonadales	Succinivibrionaceae	Succinivibrio
OTU_900	Firmicutes	Clostridia	Clostridiales	Ruminococcaceae	Sporobacter
OTU_933	Firmicutes	Erysipelotrichia	Erysipelotrichales	Erysipelotrichaceae	Clostridium XVIII
OTU_934	Proteobacteria	Betaproteobacteria	Burkholderiales	Comamonadaceae	Delftia
OTU_991	Bacteroidetes	Bacteroidia	Bacteroidales	Prevotellaceae	Prevotella
OTU_1002	Firmicutes	Clostridia	Clostridiales	Ruminococcaceae	Anaerotruncus
OTU_1057	Firmicutes	Clostridia	Clostridiales	Lachnospiraceae	Fusicatenibacter
OTU_1077	Firmicutes	Clostridia	Clostridiales	Lachnospiraceae	Clostridium XlVa
OTU_1100	Proteobacteria	Gammaproteobacteria	Xanthomonadales	Xanthomonadaceae	Stenotrophomonas
OTU_1118	Bacteroidetes	Bacteroidia	Bacteroidales	Prevotellaceae	Prevotella
OTU_1140	Bacteroidetes	Bacteroidia	Bacteroidales	Prevotellaceae	Prevotella
OTU_1160	Bacteroidetes	Bacteroidia	Bacteroidales	Prevotellaceae	Prevotella
OTU_1168	Bacteroidetes	Bacteroidia	Bacteroidales	Bacteroidaceae	Bacteroides
OTU_1182	Bacteroidetes	Bacteroidia	Bacteroidales	Prevotellaceae	Prevotella
OTU_1217	Bacteroidetes	Bacteroidia	Bacteroidales	Bacteroidaceae	Bacteroides
OTU_1246	Firmicutes	Clostridia	Clostridiales	Lachnospiraceae	Clostridium XlVa
OTU_1251	Bacteroidetes	Bacteroidia	Bacteroidales	Porphyromonadaceae	Butyricimonas
OTU_1252	Bacteroidetes	Bacteroidia	Bacteroidales	Porphyromonadaceae	Coprobacter
OTU_1253	Firmicutes	Clostridia	Clostridiales	Ruminococcaceae	Clostridium IV
